# Evaluation of Frequency of Administration of Intravenous Bisphosphonate and Recurrent Skeletal-Related Events in Patients With Multiple Myeloma

**DOI:** 10.1001/jamanetworkopen.2021.18410

**Published:** 2021-07-27

**Authors:** Jifang Zhou, Karen Sweiss, Jin Han, Naomi Y. Ko, Pritesh R. Patel, Brian C.-H. Chiu, Gregory S. Calip

**Affiliations:** 1 Department of Pharmacy Systems, Outcomes and Policy, University of Illinois at Chicago, Chicago; 2 School of International Pharmaceutical Business, China Pharmaceutical University, Nanjing, China; 3 Department of Pharmacy Practice, University of Illinois at Chicago, Chicago; 4 Section of Hematology Oncology, Boston University School of Medicine, Boston, Massachusetts; 5 Division of Hematology and Oncology, University of Illinois at Chicago, Chicago; 6 Department of Public Health Sciences, The University of Chicago, Chicago, Illinois

## Abstract

This cohort study examines the risk of skeletal-related events associated with the frequency of bisphosphonate treatment in patients with multiple myeloma.

## Introduction

Intravenous bisphosphonates have demonstrated efficacy in preventing the occurrence of skeletal-related events (SREs),^1^ defined clinically by hypercalcemia, spinal cord compression, pathological fracture, and other conditions requiring surgery or radiotherapy to the affected bone. Bisphosphonates were the only treatment indicated for symptomatic bone disease in patients with multiple myeloma (MM) prior to the approval of denosumab in 2018.^2^ Dosing interval studies have resulted in changes in clinical practice that de-escalate or extend the frequency of treatment from the recommended schedule of every 4 weeks to every 8 or 12 weeks^3^; however, these studies mostly included patients with solid tumors with bone metastases vs patients with MM with osteolytic bone disease, and they typically evaluated risks of a first-time SRE, excluding information on recurrences. To address these knowledge gaps, we investigated the association between bisphosphonate administration frequency with risks of recurrent SREs.

## Methods

This retrospective cohort analysis using the IBM MarketScan Databases from January 1, 2009, through September 30, 2015, was approved by the University of Illinois at Chicago institutional review board. The requirement for informed consent was waived because these analyses of deidentified data did not meet the definition of human participant research, as defined by 45 CFR 46.102(e), 21 CFR 50.3(g), and 21 CFR 56.102(e). Data analyses were conducted between September 2019 and February 2020. The Strengthening the Reporting of Observational Studies in Epidemiology (STROBE) reporting guideline for cohort studies was used in reporting this study. Following a 6-month delayed entry period, eligible patients ages 18 years and older with newly diagnosed MM and who initiated an intravenous (IV) bisphosphonate therapy (pamidronate, zoledronic acid, or both) regimen were included in the analytical cohort. Time-varying bisphosphonate administration frequency was defined using recommended intervals of every 4 weeks or delayed intervals of 5 to 8 weeks, 9 to 12 weeks, or longer than 12 weeks, assessed in consecutive 60-day windows after bisphosphonate initiation. The primary outcome of interest was recurrent SRE, defined as spinal cord compression, pathological fracture, or other condition requiring radiotherapy or surgery to the bone, assessed using diagnosis and procedure administrative claims codes.

Adjusted hazard ratios (HRs) and 95% CIs were calculated using robust estimators and SEs. We estimated the association of bisphosphonate use patterns with risk of first SRE with Andersen-Gill models^4^ and with risk of subsequent recurrent SRE with Prentice-Williams-Peterson models,^5^ with adjustment for measured confounders and time-dependent treatment. We used 21-day windows in cluster-related SRE claims from the same episode to identify recurrent SREs occurring independently of previous events; robust sandwich covariance matrix estimates were calculated to account for intracluster dependence of at-risk periods from the same individual. All tests were 2-tailed and considered to be statistically significant at *P* < .001 to maintain a family-wise type I error rate of .05 accounting for multiple comparisons (≤50). Data were analyzed using SAS statistical software version 9.4 (SAS Institute).

## Results

We identified 4281 eligible patients with MM, with a mean (SD) age at diagnosis of 64.8 (11.5) years, and 2452 (57.3%) were men. The median (interquartile range [IQR]) follow-up was 13.3 (8.8-17.3) months from MM diagnosis. Of 4281 patients who began IV bisphosphonate treatment, 2567 (60%) experienced at least 1 SRE (Table 1). During follow-up, we identified a total of 3345 SREs during IV bisphosphonate therapy, and in the 180 days after treatment discontinuation, the overall SRE rate was 96.2 (95% CI, 93.0-99.5) events per 100 person-years (Table 2). Adjusted Andersen-Gill risk estimates for the longer than 12 weeks (HR, 0.90; 95% CI, 0.75-1.09), 5 to 8 weeks (HR, 1.00; 95% CI, 0.89-1.13) , and 9 to 12 weeks (HR, 0.95; 95% CI, 0.84-1.08) dosing schedules were not statistically significant compared with the every 4 weeks dosing schedule. No significant or clinically meaningful differences were found in risks of first and subsequent SREs in Prentice-Williams-Peterson models.

**Table 1.** Descriptive Characteristics of Patients with MM Initiating Intravenous Bisphosphonates by Baseline SRE Status

Characteristic	Patients, No. (%)			*P* value^a^
	All (N = 4281)	With baseline SRE (n = 2567)	Without baseline SRE (n = 1714)	
Age, y				
Mean (SD)	64.8 (11.5)	64.8 (11.5)	64.8 (11.6)	.89
<50	357 (8.3)	215 (8.4)	142 (8.3)	.98
50-64	2010 (47.0)	1202 (46.8)	808 (47.1)	
≥65	1914 (44.7)	1150 (44.8)	764 (44.6)	
Sex				
Men	2452 (57.3)	1480 (57.7)	972 (56.7)	.54
Women	1829 (42.7)	1087 (42.3)	742 (43.3)	
Year of MM diagnosis				
2009	9 (0.2)	6 (0.2)	3 (0.2)	<.001
2010	814 (19.0)	486 (18.9)	328 (19.1)	
2011	888 (20.7)	576 (22.4)	312 (18.2)	
2012	922 (21.5)	574 (22.4)	348 (20.3)	
2013	745 (17.4)	466 (18.2)	279 (16.3)	
2014	634 (14.8)	364 (14.2)	270 (15.8)	
2015	269 (6.3)	95 (3.7)	174 (10.2)	
History of ED visits	2040 (47.7)	1281 (49.9)	759 (44.3)	<.001
History of hospitalization	1013 (23.7)	620 (24.2)	393 (22.9)	.36
Comorbid conditions				
Falls	131 (3.1)	90 (3.5)	41 (2.4)	.04
Gait abnormalities	153 (3.6)	104 (4.1)	49 (2.9)	.04
Syncope	157 (3.7)	92 (3.6)	65 (3.8)	.72
Alzheimer disease	15 (0.4)	8 (0.3)	7 (0.4)	.60
Parkinson disease	34 (0.8)	24 (0.9)	10 (0.6)	.20
Stroke or transient ischemic attack	155 (3.6)	99 (3.9)	56 (3.3)	.31
Asthma or COPD	620 (14.5)	396 (15.4)	224 (13.1)	.03
Heart failure	234 (5.5)	139 (5.4)	95 (5.5)	.86
Major depressive disorder	94 (2.2)	64 (2.5)	30 (1.8)	.10
Diabetes	953 (22.3)	593 (23.1)	360 (21.0)	.11
Non-ESKD kidney disease	402 (9.4)	243 (9.5)	159 (9.3)	.84
Anemia	1679 (39.2)	949 (37.0)	730 (42.6)	<.001
Osteoporosis	485 (11.3)	325 (12.7)	160 (9.3)	<.001
Rheumatoid arthritis	67 (1.6)	45 (1.8)	22 (1.3)	.23
NCI-CCI score				
0-2	1185 (27.7)	686 (26.7)	499 (29.1)	.22
3-4	1643 (38.4)	989 (38.5)	654 (38.2)	
≥5	1453 (33.9)	892 (34.7)	561 (32.7)	
MM treatments received				
Bisphosphonates				
Zoledronic acid only	3577 (83.6)	2135 (83.2)	1442 (84.1)	.003
Pamidronate only	411 (9.6)	231 (9.0)	180 (10.5)	
Zoledronic acid and pamidronate	293 (6.8)	201 (7.8)	92 (5.4)	
Cytotoxic chemotherapy	1085 (25.3)	698 (27.2)	387 (22.6)	<.001
IMIDs	2598 (60.7)	1619 (63.1)	979 (57.1)	<.001
Proteasome inhibitors	3489 (81.5)	2132 (83.1)	1357 (79.2)	.001
Autologous HSCT	1372 (32.0)	886 (34.5)	486 (28.4)	<.001

Abbreviations: COPD, chronic obstructive pulmonary disease; ED, emergency department; ESKD, end-stage kidney disease; HSCT, hematopoietic stem cell transplantation; IMID, immunomodulatory imide drug; MM, multiple myeloma; NCI-CCI, National Cancer Institute Charlson Comorbidity Index; SRE, skeletal related events.

^a^ To compare groups we used *t* test for comparison of means and χ^2^ test for categorical variables.

**Table 2.** Rates and Risk of First and Recurrent of SREs Associated With Cumulative Administration Schedule of Intravenous Bisphosphonates

Analysis	Time from bisphosphonate initiation, d					Total	Univariate analysis		Multivariable analysis^a^	
	61-180	181-360	361-540	541-720	≥721		HR (95% CI	*P* value	HR (95% CI	*P* value
Overall										
SREs, No.	1381	1041	439	214	270	3345	NA	NA	NA	NA
Person-years	595.4	1348.7	682.9	375.7	474.3	3477	NA	NA	NA	NA
SRE rate, per 100 person-years	231.9 (219.9-244.4)	77.2 (72.6-82.0)	64.3 ()58.5-70.5	57.0 (49.7-64.9)	56.9 (50.4-64.0)	96.2 (93.0-99.5)	NA	NA	NA	NA
Bisphosphonate treatment every 4 wk										
SREs, No.	282	28	2	1	0	313	NA	NA	NA	NA
Person-years	147.1	67.2	6.5	1.6	0.7	223.1	NA	NA	NA	NA
SRE rate, per 100 person-years	191.7 (170.2-215.0)	41.6 (28.1-59.1)	30.8 (5.1-95.0)	63.7 (3.6-274.9)	NA	140.3 (125.3-156.4)	NA	NA	NA	NA
Risk of first SRE^b^	NA	NA	NA	NA	NA	NA	1 [Reference]	NA	1 [Reference]	NA
Risk of recurrent SRE^c^										
First	NA	NA	NA	NA	NA	NA	1 [Reference]	NA	1 [Reference]	NA
Second	NA	NA	NA	NA	NA	NA	1 [Reference]	NA	1 [Reference]	NA
Third	NA	NA	NA	NA	NA	NA	1 [Reference]	NA	1 [Reference]	NA
Bisphosphonate treatment every 5-8 wk										
SREs, No.	749	539	311	159	220	1978	NA	NA	NA	NA
Person-years	312.1	708.6	433.8	275.2	348.5	2078.2	NA	NA	NA	NA
SRE rate, per 100 person-years	240.0 (223.2-257.6)	76.1 (69.8-82.7)	71.7 (64.0-80.0)	57.8 (49.3-67.2)	63.1 (55.1-71.8)	95.2 (91.0-99.4)	NA	NA	NA	NA
Risk of first SRE^b^	NA	NA	NA	NA	NA	NA	1.00 (0.89-1.12)	.95	1.00 (0.89-1.12)	.95
Risk of recurrent SRE^c^										
First	NA	NA	NA	NA	NA	NA	0.98 (0.85-1.12)	.75	0.98 (0.85-1.12)	.75
Second	NA	NA	NA	NA	NA	NA	1.17 (0.79-1.73)	.43	1.17 (0.79-1.73)	.43
Third	NA	NA	NA	NA	NA	NA	0.82 (0.34-1.97)	.66	0.82 (0.34-1.97)	.66
Bisphosphonate treatment every 9-12 wk										
SREs, No.	288	362	115	51	49	865	NA	NA	NA	NA
Person-years	127.9	397.9	214.3	92.1	120.6	952.8	NA	NA	NA	NA
SRE rate, per 100 person-years	225.2 (200.1-252.2)	91.0 (81.9-100.7)	53.7 (44.4-64.1)	55.4 (41.5-72.0)	40.6 (30.3-53.1)	90.8 (84.9-97.0)	NA	NA	NA	NA
Risk of first SRE^b^	NA	NA	NA	NA	NA	NA	0.95 (0.84-1.08)	.41	0.95 (0.84-1.08)	.46
Risk of recurrent SRE^c^										
First	NA	NA	NA	NA	NA	NA	0.96 (0.82-1.12)	.62	0.98 (0.84-1.15)	.80
Second	NA	NA	NA	NA	NA	NA	1.11 (0.74-1.66)	.62	1.15 (0.76-1.73)	.52
Third	NA	NA	NA	NA	NA	NA	0.72 (0.30-1.71)	.45	0.85 (0.31-2.34)	.75
Bisphosphonate treatment interval >12 wk										
SREs, No.	62	112	11	3	1	189	NA	NA	NA	NA
Person-years	8.3	175.0	28.3	6.8	4.5	222.9	NA	NA	NA	NA
SRE rate, per 100 person-years	747.9 (576.1-948.7)	64.0 (52.9-76.6)	38.9 (20.2-66.6)	44.4 (11.0-114.4)	22.0 (1.3-97.7)	84.8 (73.3-97.5)	NA	NA	NA	NA
Risk of first SRE^b^	NA	NA	NA	NA	NA	NA	0.88 (0.73-1.05)	.16	0.90 (0.75-1.09)	.29
Risk of recurrent SRE^c^										
First	NA	NA	NA	NA	NA	NA	0.80 (0.62-1.02)	.08	0.84 (0.65-1.08)	.17
Second	NA	NA	NA	NA	NA	NA	1.19 (0.76-1.85)	.45	1.23 (0.79-1.94)	.36
Third	NA	NA	NA	NA	NA	NA	0.79 (0.29-2.11)	.63	0.95 (0.31-2.92)	.93

Abbreviations: HR, hazard ratio; NA, not applicable; SRE, skeletal-related event.

^a^ Adjusted for patient demographics, baseline comorbid conditions and health care utilization, and time-varying treatment with chemotherapy, immunomodulatory imide drugs, proteasome inhibitors, and autologous HSCT.

^b^ Calculated using an Anderson-Gill model.

^c^ Calculated using a Prentice-Williams-Peterson total time model.

## Discussion

The findings of this cohort study agree with findings from randomized clinical trials showing that less frequent IV bisphosphonate administration frequency was associated with comparable recurrent SRE risk reductions as more frequent administration.^6^ In clinical practice, there are many reasons why oncologists administer IV bisphosphonates at longer dosing intervals, and additional attention should consider the potential benefits and risks of more or less frequent bone-directed therapy. One notable strength of our study was that we used a robust approach to assess first and recurrent SREs from the date of IV bisphosphonate initiation through 180 days after treatment discontinuation. However, our findings should be interpreted considering its limitations: in this observational study, misclassification and unmeasured confounding cannot be ruled out.

Multiple reasons exist for delays or disparities in supportive care with IV bisphosphonates, including adverse events, such as osteonecrosis of the jaw, and other circumstances compromising the safety of supportive cancer care, such as the COVID-19 pandemic. In line with other clinical studies that primarily include patients with bone metastases, our findings support the relative safety, in terms of SRE risk, among patients with MM when the frequency of bisphosphonate therapy is reduced.

## References

[1] Berenson JR, Lichtenstein A, Porter L, ; Myeloma Aredia Study Group. Efficacy of pamidronate in reducing skeletal events in patients with advanced multiple myeloma. N Engl J Med. 1996;334(8):488-493. doi:10.1056/NEJM199602223340802 8559201

[2] Kumar SK, Callander NS, Alsina M, . Multiple myeloma, version 3.2017, NCCN clinical practice guidelines in oncology. J Natl Compr Canc Netw. 2017;15(2):230-269. doi:10.6004/jnccn.2017.0023 28188192

[3] Awan AA, Paterson A, Clemons M. De-escalation of bone-modifying agents in patients with bone metastases: the best of times and the worst of times? J Oncol Pract. 2018;14(8):465-467. doi:10.1200/JOP.18.00393 30096270

[4] Andersen PK, Gill RD. Cox's regression model for counting processes: a large sample study. Ann Statist. 1982;10(4):1100-1120. doi:10.1214/aos/1176345976

[5] Prentice RL, Williams BJ, Peterson AV. On the regression analysis of multivariate failure time data. Biometrika. 1981;68(2):373-379. doi:10.1093/biomet/68.2.373

[6] Himelstein AL, Foster JC, Khatcheressian JL, . Effect of longer-interval vs standard dosing of zoledronic acid on skeletal events in patients with bone metastases: a randomized clinical trial. JAMA. 2017;317(1):48-58. doi:10.1001/jama.2016.19425 28030702 PMC5321662

